# Flexibilins A–C, New Cembrane-Type Diterpenoids from the Formosan Soft Coral, *Sinularia flexibilis*

**DOI:** 10.3390/md11061999

**Published:** 2013-06-10

**Authors:** Li-Chung Hu, Jui-Hsin Su, Michael Yen-Nan Chiang, Mei-Chin Lu, Tsong-Long Hwang, Yung-Husan Chen, Wan-Ping Hu, Nai-Cheng Lin, Wei-Hsien Wang, Lee-Shing Fang, Yueh-Hsiung Kuo, Ping-Jyun Sung

**Affiliations:** 1 Graduate Institute of Marine Biotechnology and Department of Life Science and Institute of Biotechnology, National Dong Hwa University, Pingtung 944, Taiwan; E-Mails: stoja582@gmail.com (L.-C.H.); x2219@nmmba.gov.tw (J.-H.S.); jinx6609@nmmba.gov.tw (M.-C.L.); 2 National Museum of Marine Biology and Aquarium, Pingtung 944, Taiwan; E-Mails: tony_chen72001@yahoo.com.tw (Y.-H.C.); lnc7222@hotmail.com (N.-C.L.); whw@nmmba.gov.tw (W.-H.W.); 3 Department of Marine Biotechnology and Resources and Asia-Pacific Ocean Research Center, National Sun Yat-sen University, Kaohsiung 804, Taiwan; 4 Department of Chemistry, National Sun Yat-sen University, Kaohsiung 804, Taiwan; E-Mail: michael@mail.nsysu.edu.tw; 5 Graduate Institute of Natural Products, Chang Gung University, Taoyuan 333, Taiwan; E-Mail: htl@mail.cgu.edu.tw; 6 Department of Biotechnology, Kaohsiung Medical University, Kaohsiung 807, Taiwan; E-Mail: wphu@kmu.edu.tw; 7 Department of Sport, Health and Leisure, Cheng Shiu University, Kaohsiung 833, Taiwan; E-Mail: lsfang@csu.edu.tw; 8 Tsuzuki Institute for Traditional Medicine, China Medical University, Taichung 404, Taiwan; 9 Graduate Institute of Natural Products, Kaohsiung Medical University, Kaohsiung 807, Taiwan

**Keywords:** cembrane, diterpenoid, *Sinularia flexibilis*, elastase

## Abstract

Three new cembrane-type diterpenoids, flexibilins A–C (**1**–**3**), along with a known cembrane, (−)-sandensolide (**4**), were isolated from the soft coral, *Sinularia flexibilis*. The structures of cembranes **1**–**4** were elucidated by spectroscopic methods. The structure of **4**, including its absolute stereochemistry, was further confirmed by single-crystal X-ray diffraction analysis. Cembrane **2** displayed a moderate inhibitory effect on the release of elastase by human neutrophils.

## 1. Introduction

**Figure 1:**
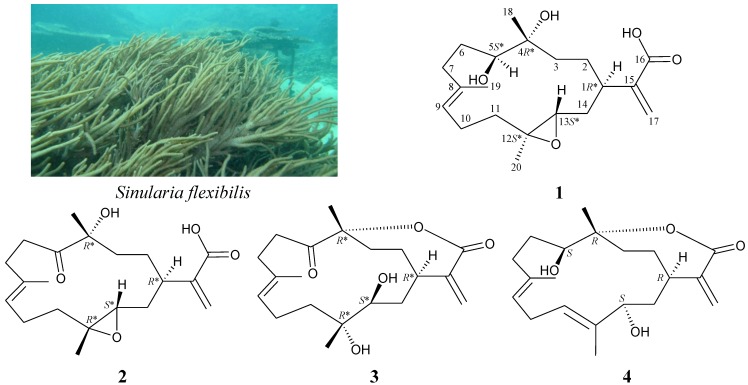
The soft coral, *Sinularia flexibilis*, and the structures of flexibilins A–C (**1**–**3**) and (−)-sandensolide (**4**).

Octocorals, particularly, soft corals belonging to the genus *Sinularia*, have been demonstrated to be rich sources of bioactive natural products [1,2]. Previous chemical investigations on *Sinularia flexibilis*, an octocoral distributed widely in the tropical and subtropical waters of the Indo-Pacific Ocean, have yielded a series of interesting cembrane-type diterpenoids [3,4,5,6,7,8,9,10,11,12,13,14,15,16,17,18,19,20], and most of these compounds have been shown to possess various bioactivities, such as cytotoxic [4,6,11,12,13,14,15,16,17,20,21], anti-inflammatory [19,20], neuroprotective [19] and algicidal [9] effects. In continuation of our search for new substances from marine invertebrates collected from the waters of Taiwan, the octocoral *Sinularia flexibilis* (Quoy and Gaimard, 1833) was studied (Figure 1), as its organic extract was found to display meaningful signals in NMR studies. Three new cembrane-type diterpenoids, flexibilins A–C (**1**–**3**), and a known cembrane, (−)-sandensolide (**4**) [10,22,23], were isolated (Figure 1). In this paper, we reported the isolation, structure determination and bioactivity of cembranes **1**–**4**.

## 2. Results and Discussion

**Figure 2:**
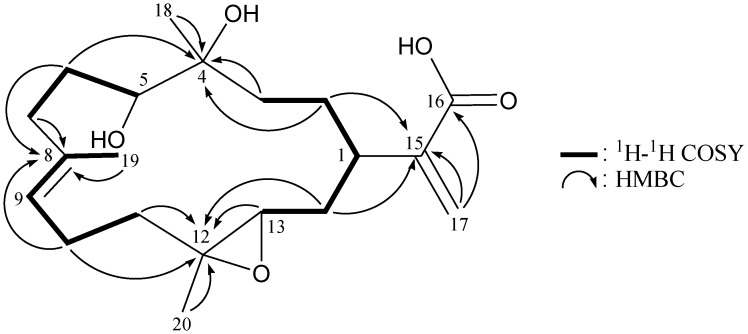
^1^H–^1^H COSY and selected HMBC correlations (protons→quaternary carbons) for cembrane **1**.

Flexibilin A (**1**) was obtained as yellowish oil, and its molecular formula, C_20_H_32_O_5_, was determined according to a pseudomolecular ion [M + Na]^+^ at *m/z* 375.2144 (calcd. for C_20_H_32_O_5_Na, 375.2147) identified by HRESIMS, as well as ^13^C NMR coupled with DEPT spectra (Table 1), which indicated that the double bond equivalence (DBE) of **1** was five. The IR spectrum of **1** revealed the presence of carboxylic acid, hydroxy and ester functionalities from absorptions at 3750–2400, 3419 and 1711 cm^−1^, respectively. The ^13^C NMR data of **1** showed the presence of 20 carbon signals in total, which were assigned by the assistance of the DEPT spectrum to three methyls, seven sp^3^ methylenes, three sp^3^ methines, two sp^3^ quaternary carbons, an sp^2^ methylene, an sp^2^ methine and three sp^2^ quaternary carbons. ^13^C NMR signals appearing at *δ*_C_ 170.8 (C-16), 142.7 (C-15) and 126.5 (CH_2_-17) and proton NMR signals appearing at *δ*_H_ 6.40 (1H, s) and 5.68 (1H, s) suggested the presence of a α-exomethylene-carboxylic acid moiety. The main carbon skeleton of **1** was elucidated by ^1^H–^1^H COSY and HMBC experiments (Table 1 and Figure 2). From the ^1^H–^1^H COSY spectrum of **1**, it was possible to establish the separate spin systems of H-13/H_2_-14/H-1/H_2_-2/H_2_-3, H-5/H_2_-6/H_2_-7, H-9/H_2_-10/H_2_-11 and H-9/H_3_-19 (by allylic coupling). These data, together with the key HMBC correlations between protons and quaternary carbons, such as H_2_-2, H_2_-3, H_2_-6, H_3_-18/C-4; H_2_-6, H_2_-7, H_2_-10, H_3_-19/C-8; H_2_-10, H_2_-11, H-13, H_2_-14, H_3_-20/C-12; H_2_-2, H_2_-14, H_2_-17/C-15; and H_2_-17/C-16, permitted elucidation of the main carbon skeleton of **1**. The tertiary methyls at C-4, C-8 and C-12 were confirmed by HMBC correlations between H_3_-18/C-3, -4, -5; H_3_-19/C-7, -8, -9; and H_3_-20/C-11, -12, -13. Thus, from the reported data, the skeleton of **1** was identified as a cembrane-type diterpenoid with two rings.

**Table 1:** ^1^H (500 MHz, CDCl_3_) and ^13^C (125 MHz, CDCl_3_) NMR data and ^1^H–^1^H COSY and HMBC correlations for cembrane **1**.

Position	*δ*_H_ (*J* in Hz)	*δ*_C_, Multiple	^1^H–^1^H COSY	HMBC
1	2.70 br s	38.1, CH	H_2_-2, H_2_-14	C-13
2	1.63 m; 1.52 m	26.6, CH_2_	H-1, H_2_-3	C-1, -4, -14, -15
3	1.60 m; 1.53 m	37.1, CH_2_	H_2_-2	C-2, -4, -5
4		74.8, C		
5	3.53 d (10.0)	74.0, CH	H_2_-6	C-6, -7, -18
6	1.71 m; 1.59 m	28.6, CH_2_	H-5, H_2_-7	C-4, -5, -7, -8
7	2.21 m; 2.14 m	33.7, CH_2_	H_2_-6	C-5, -6, -8, -9, -19
8		136.1, C		
9	5.11 dd (6.5, 6.5)	122.7, CH	H_2_-10, H_3_-19	C-7, -10, -11, -19
10	2.14 m	23.1, CH_2_	H-9, H_2_-11	C-8, -9, -11, -12
11	2.01 ddd (14.0, 6.5, 3.0)	37.5, CH_2_	H_2_-10	C-9, -10, -12, -20
	1.42 ddd (14.0, 11.0, 3.0)			
12		60.3, C		
13	2.87 dd (7.5, 5.5)	61.2, CH	H_2_-14	C-11, -12, -14
14	1.85 ddd (14.0, 10.0, 5.5); 1.61 m	34.1, CH_2_	H-1, H-13	C-1, -2, -12, -13, -15
15		142.7, C		
16		170.8, C		
17	6.40 s; 5.68 s	126.5, CH_2_		C-1, -15, -16
18	1.26 s	24.7, CH_3_		C-3, -4, -5
19	1.67 s	18.1, CH_3_	H-9	C-7, -8, -9
20	1.30 s	17.1, CH_3_		C-11, -12, -13

**Figure 3:**
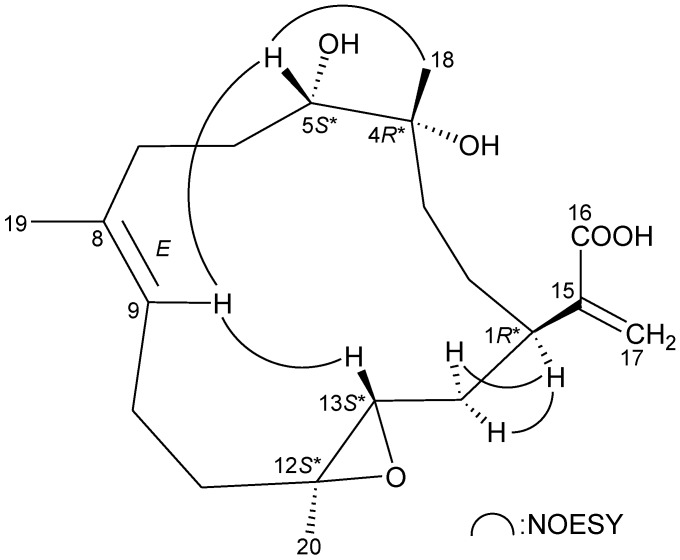
Key NOESY correlations of **1**.

The relative configuration of **1** was elucidated from the interactions observed in a NOESY experiment, as shown in Figure 3. In the NOESY experiment of **1**, H-1 was found to be correlated with H_2_-14, but not with H-13; this, plus, the lack of correlation between H-13 and H_3_-20 demonstrated that H-1, H-13 and Me-20 were α-, β- and α-oriented, respectively. Additionally, correlations between H-9/H-5 and H-9/H-13, and the absence of correlation between H-9/H_3_-19, reflected the *E* geometry of the double bond at C-8/9. From modeling analysis, H-9 was found to be close to H-5 and H-13, when H-5 and H-13 were β-oriented. H_3_-18 was found to be correlated with H-5, but not with H-1, indicating that the 4-hydroxy group was α-oriented. Based on the above findings, the structure of **1** was elucidated, and the chiral carbons of **1** were assigned as 1R*, 4R*, 5S*, 12S* and 13S*. 

**Figure 4:**
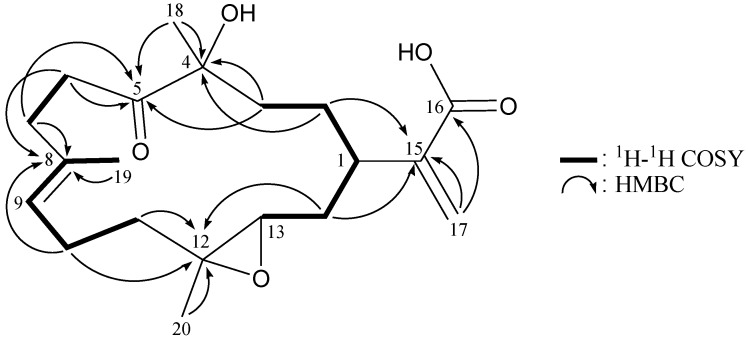
^1^H–^1^H COSY and selected HMBC correlations (protons→quaternary carbons) for cembrane **2**.

The new cembrane diterpene, flexibilin B (**2**), had the molecular formula, C_20_H_30_O_5_, as deduced by HRESIMS (*m/z* calcd.: 373.1991; found 373.1989 [M + Na]^+^) (6° of unsaturation). The IR spectrum of **2** revealed the presence of carboxylic acid (ν_max_ 3700–2400 cm^−1^), hydroxy (ν_max_ 3447 cm^−1^) and carbonyl (ν_max_ 1713 cm^−1^) moieties. From the ^13^C NMR and DEPT spectra of **2** (Table 2), a trisubstituted olefin (*δ*_C_ 134.5, C-8; 126.5, CH-9), a α-exomethylene-carboxylic acid (*δ*_C_ 171.3, C-16; 141.9, C-15; 126.2, CH_2_-17), a keto carbonyl (*δ*_C_ 213.6, C-5) and a trisubstituted epoxide (*δ*_C_ 60.8, C-12; 59.4, CH-13) were observed. The coupling information in the ^1^H–^1^H COSY spectrum of **2** enabled identification of the H-13/H_2_-14/H-1/H_2_-2/H_2_-3, H_2_-6/H_2_-7, H-9/H_2_-10/H_2_-11 and H-9/H_3_-19 (by allylic coupling) units. From these data, together with the results of an HMBC experiment for **2 **(Table 2 and Figure 4), the molecular framework of **2** could be established. 

**Table 2:** ^1^H (500 MHz, CDCl_3_) and ^13^C (125 MHz, CDCl_3_) NMR data and ^1^H–^1^H COSY and HMBC correlations for cembrane **2**.

Position	*δ*_H_ (*J* in Hz)	*δ*_C_, Multiple	^1^H–^1^H COSY	HMBC
1	2.75 m	35.9, CH	H_2_-2, H_2_-14	C-14
2	1.59 m; 1.41 m	25.1, CH_2_	H-1, H_2_-3	C-4, -15
3	1.78 m; 1.52 m	35.8, CH_2_	H_2_-2	C-4, -5, -18
4		78.9, C		
5		213.6, C		
6	2.70 m	34.2, CH_2_	H_2_-7	C-5, -7, -8
7	2.55 m; 2.21 m	31.5, CH_2_	H_2_-6	C-5, -6, -8, -9, -19
8		134.5, C		
9	5.13 dd (6.0, 6.0)	126.5, CH	H_2_-10, H_3_-19	C-7, -10, -11, -19
10	2.10 m; 2.01 m	22.8, CH_2_	H-9, H_2_-11	C-8, -9, -11, -12
11	1.96 m; 1.56 m	36.7, CH_2_	H_2_-10	C-9, -10, -12, -13
12		60.8, C		
13	2.79 dd (9.5, 4.0)	59.4, CH	H_2_-14	C-14
14	1.99 m; 1.42 m	32.4, CH_2_	H-1, H-13	C-1, -12, -13, -15
15		141.9, C		
16		171.3, C		
17	6.42, s; 5.57 s	126.2, CH_2_		C-1, -15, -16
18	1.35 s	25.7, CH_3_		C-3, -4, -5
19	1.66 s	17.0, CH_3_	H-9	C-7, -8, -9
20	1.27 s	18.2, CH_3_		C-11, -12, -13

**Figure 5:**
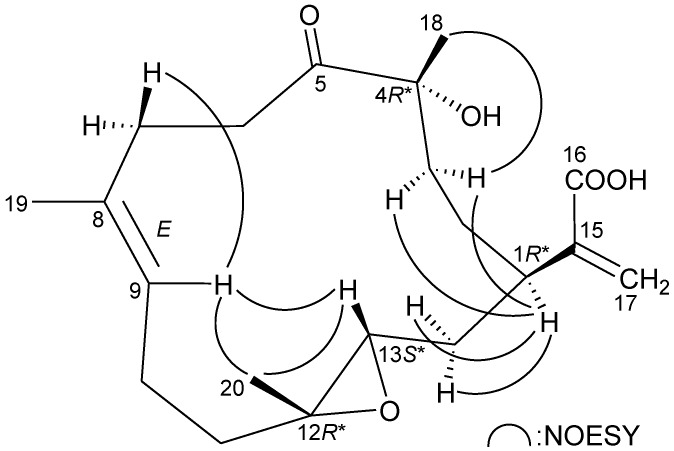
Key NOESY correlations of **2**.

The relative stereochemistry of **2** was elucidated from the interactions observed in a NOESY experiment (Figure 5). Due to the α-orientation of H-1, the epoxy proton, H-13, was identified as being β-oriented, as no correlation was observed between H-1 and H-13. H-9 displayed correlations with H-13 and one of the C-7 methylene protons (*δ*_H_ 2.55), and there was a lack of correlation between H-9 and H_3_-19, which reflects the *E* geometry of the double bond between C-8/9. H_3_-20 showed correlations with H-9 and H-13, indicating that Me-20 is of a β-orientation at C-12. Furthermore, H-1 was found to be correlated with H_2_-3, and H_3_-18 exhibited a correlation with one of the C-3 methylene protons (*δ*_H_ 1.78), which indicated the β-orientation of Me-18 at C-4 by molecular modeling analysis. Based on the above information, the chiral carbons of cembrane **2** were assigned as 1R*, 4R*, 12R* and 13S*.

**Figure 6:**
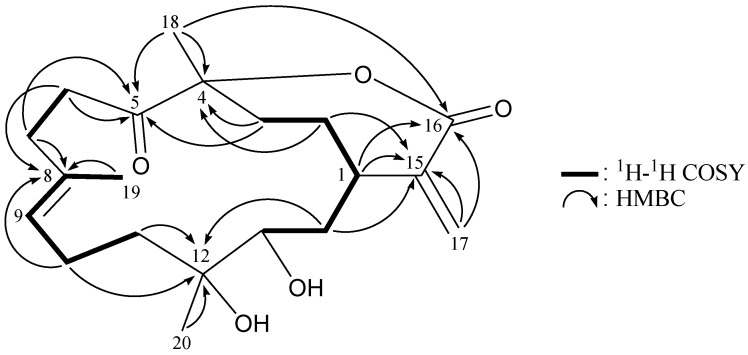
^1^H–^1^H COSY and selected HMBC correlations (protons→quaternary carbons) for cembrane **3**.

Flexibilin C (**3**) was obtained as a white powder. The molecular formula of **3 **was established as C_20_H_30_O_5_ (6° of unsaturation) from a sodiated molecule at *m/z* 373 in the ESIMS spectrum and further supported by HRESIMS (*m/z* 373.1989, calcd. for C_20_H_30_O_5_Na, 373.1991). The IR spectrum of **3** exhibited the presence of hydroxy (ν_max_ 3431 cm^−1^) and carbonyl (ν_max_ 1711 cm^−1^) groups. From the ^13^C NMR data of **3** (Table 3), a suite of resonances at *δ*_C_ 168.0 (C-16), 143.3 (C-15), 126.0 (CH_2_-17), 90.0 (C-4), 37.4 (CH-1), 33.7 (CH_2_-3) and 29.7 (CH_2_-2) could be assigned to the α-exomethylene-ε-lactone moiety. Two additional unsaturated functionalities were indicated by ^13^C resonances at *δ*_C_ 209.3 (C-5), 134.7 (C-8) and 124.9 (CH-9), suggesting the presence of a keto carbonyl and a trisubstituted olefin. On the basis of the overall unsaturation data, **3** was concluded to be a diterpenoid molecule possessing two rings. The ^1^H NMR spectrum of **3** showed the presence of three methyl groups: two singlets at *δ*_H_ 1.27 and 1.49, representing the methyl groups on oxygenated quaternary carbons, respectively, and a vinyl methyl at *δ*_H_ 1.69. The ^1^H NMR coupling information in the ^1^H–^1^H COSY spectrum of **3** enabled identification of the H-13/H_2_-14/H-1/H_2_-2/H_2_-3, H_2_-6/H_2_-7, H-9/H_2_-10/H_2_-11 and H-9/H_3_-19 (by allylic coupling) units, which were assembled with the assistance of an HMBC experiment (Table 3 and Figure 6). The key HMBC correlations between protons and quaternary carbons of **3**, such as H_2_-2, H_2_-3, H_3_-18/C-4; H_2_-3, H_2_-6, H_2_-7, H_3_-18/C-5; H_2_-6, H_2_-7, H_2_-10, H_3_-19/C-8; H_2_-10, H_2_-11, H_2_-14, H_3_-20/C-12; H-1, H_2_-2, H_2_-14, H_2_-17/C-15; and H-1, H_2_-17, H_3_-18/C-16, enabled establishment of the main carbon skeleton of **3**. A vinyl methyl at C-8 was confirmed by the allylic coupling between H-9/H_3_-19 in the ^1^H–^1^H COSY spectrum and by the HMBC correlations between H_3_-19/C-7, -8, -9. The tertiary methyls at C-4 and C-12 were confirmed by the HMBC correlations between H_3_-18/C-3, -4, -5, and H_3_-20/C-11, -12, -13. H_3_-18 showed a long-range ^4^*J*-correlation with the ester carbonyl (*δ*_C_ 168.0, C-16) in the HMBC spectrum, which further supported the existence of the ε-lactone moiety in **3**.

**Table 3:** ^1^H (500 MHz, CDCl_3_) and ^13^C (125 MHz, CDCl_3_) NMR data and ^1^H–^1^H COSY and HMBC correlations for cembrane **3**.

Position	*δ*_H_ (*J* in Hz)	*δ*_C_, Multiple	^1^H–^1^H COSY	HMBC
1	1.90 m	37.4, CH	H_2_-2, H_2_-14	C-2, -14, -15, -16, -17
2	2.19 m; 1.20 ddd (12.5, 12.5, 7.0)	29.7, CH_2_	H-1, H_2_-3	C-1, -2, -3, -4, -14, -15
3	2.45 dd (15.5, 7.0); 1.91 m	33.7, CH_2_	H_2_-2	C-1, -2, -4, -5, -18
4		90.0, C		
5		209.3, C		
6	3.36 dd (17.0, 9.5); 2.56 br d (17.0)	33.6, CH_2_	H_2_-7	C-5, -7, -8
7	2.57 br d (16.0); 2.15 m	31.0, CH_2_	H_2_-6	C-5, -6, -8, -9, -19
8		134.7, C		
9	4.99 dd (6.5, 6.5)	124.9, CH	H_2_-10, H_3_-19	C-7, -9, -10, -19
10	2.14 m	23.5, CH_2_	H-9, H_2_-11	C-8, -9, -11, -12
11	1.66 m	38.2, CH_2_	H_2_-10	C-9, -10, -12, -13, -20
12		76.2, C		
13	3.26 dd (6.0, 1.5)	75.5, CH	H_2_-14	C-1, -11, -14, -20
14	2.03 dd (14.5, 1.5)	35.3, CH_2_	H-1, H-13	C-1, -2, -12, -13, -15
	1.40 ddd (14.5, 11.0, 6.0)			
15		143.3, C		
16		168.0, C		
17	6.35 s; 5.58 s	126.0, CH_2_		C-1, -15, -16
18	1.49 s	29.6, CH_3_		C-3, -4, -5, -16
19	1.69 s	17.4, CH_3_	H-9	C-7, -8, -9
20	1.27 s	24.6, CH_3_		C-11, -12, -13

**Figure 7:**
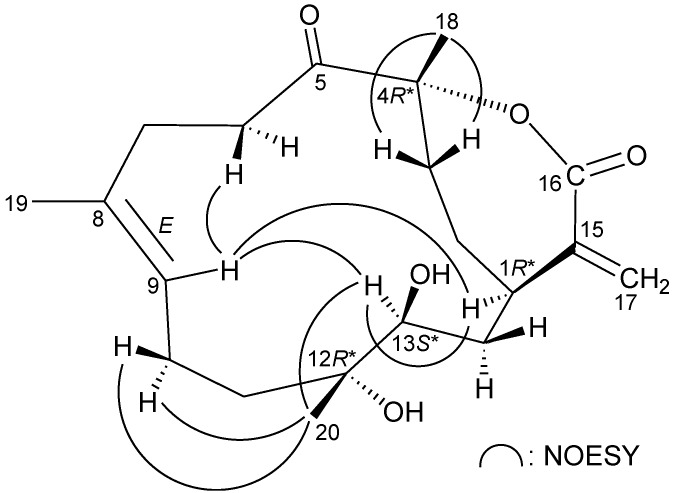
Key NOESY correlations of **3**.

The relative stereochemistry of **3** was elucidated by the analysis of NOE correlations, as shown in Figure 7. H-9 exhibited a correlation with one of the C-6 methylene protons (*δ*_H_ 3.36), but not with H_3_-19, which revealed the *E* geometry of the C-8/9 double bond. It was found that H-13 showed correlations with H-1 and H-9. From molecular modeling analysis, H-13 was found to be close to H-1 and H-9, when H-1 and H-13 were α-oriented. Correlations observed between H_3_-20/H-13 and H_3_-20/H_2_-10 reflected the β-orientation of Me-20. Furthermore, H_3_-18 exhibited correlations with C-3 methylene protons and the lack of correlation between H_3_-18 and H-1, indicating that Me-18 was positioned on the β-face in **3**. Thus, the structure of **3** was established, and the chiral carbons of **3** were assigned as 1R*, 4R*, 12R* and 13S*.

**Figure 8:**
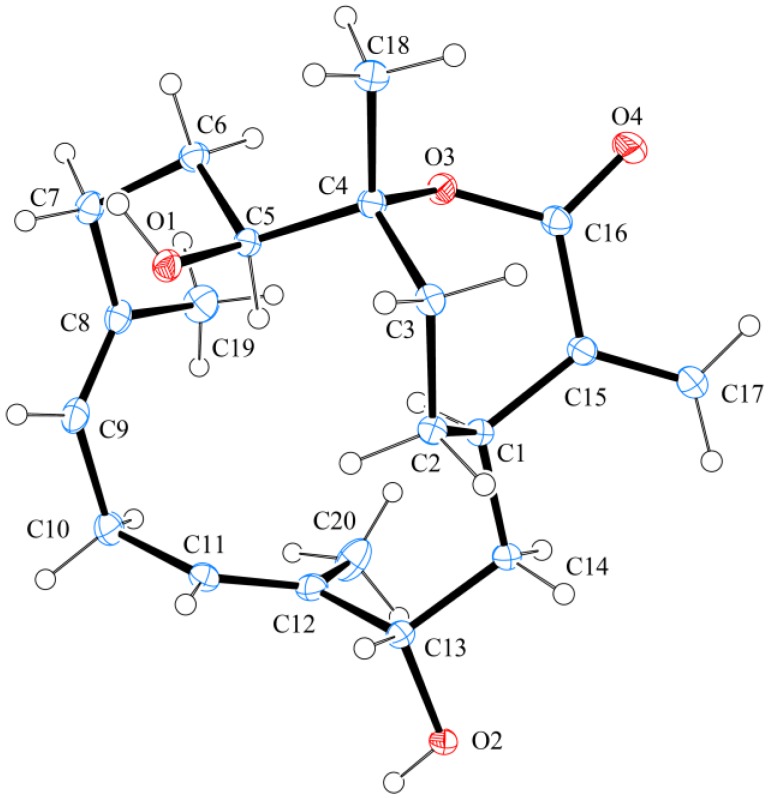
Molecular plot of **4** with confirmed absolute configuration.

The known cembrane, (−)-sandensolide (**4**), was first isolated from a Chinese soft coral, *Dendronephthya* sp. [23], and its structure was elucidated by spectroscopic methods and by comparison of spectral (1D and 2D NMR) and physical (rotation value) data with those of its enantiomer, sandensolide [10,22]. In this study, the structure of **4**, including its absolute stereochemistry, was further established by single-crystal X-ray diffraction analysis for the first time. The X-ray structure of **4** (Figure 8) demonstrates the *E* geometry of the C-8/9 and C-11/12 double bonds, and the absolute configurations for all chiral carbons were assigned as 1*R*, 4*R*, 5*S* and 13*S*. As flexibilins A–C (**1**–**3**) were isolated, along with (−)-sandensolide (**4**), from the same organism, it is reasonable on biogenetic grounds to assume that cembranes **1**–**3** have the same absolute configurations as **4**. 

The *in vitro* anti-inflammatory effects of cembranes **1**–**4** were examined, and cembrane **2** displayed a moderate inhibitory effect on the release of elastase by human neutrophils (Table 4) [24].

**Table 4:** Inhibitory effects of cembranes **1**–**4** on the generation of superoxide anions and the release of elastase by human neutrophils in response to fMLP/CB.

Compound	Superoxide anions		Elastase release	
	IC_50_ (μg/mL)	Inh% ^a^	IC_50_ (μg/mL)	Inh% ^a^
**1**	>10	12.31 ± 3.04 *	>10	22.67 ± 5.32 *
**2**	>10	22.03 ± 3.88 **	>10	45.76 ± 2.92 ***
**3**	>10	18.80 ± 3.81 **	>10	10.56 ± 2.75 *
**4**	>10	−2.08 ± 1.01	>10	8.14 ± 4.03
**LY294002 ^b^**	0.41 ± 0.27		0.79 ± 0.18	

^a ^Percentage of inhibition (Inh%) at a concentration of 10 μg/mL. ^b^ LY294002, a phosphatidylinositol-3-kinase inhibitor, was used as a positive control for inhibition of superoxide anion generation and elastase release. The results are presented as the mean ± S.E.M. (*n* = 3 or 4). * *p* < 0.05, ** *p* < 0.01, *** *p* < 0.001, compared with the control value.

## 3. Experimental Section

### 3.1. General Experimental Procedures

Optical rotations were measured on a Jasco P-1010 digital polarimeter. Infrared spectra were recorded on a Varian Diglab FTS 1000 FT-IR spectrometer; peaks are reported in cm^−1^. The NMR spectra were recorded on a Varian Inova 500 or on a Varian Mercury Plus 400 NMR spectrometer using the residual CHCl_3_ signal (*δ*_H_ 7.26 ppm) as the internal standard for ^1^H NMR and CDCl_3_ (*δ*_C_ 77.1 ppm) for ^13^C NMR. Coupling constants (*J*) are given in Hz. ESIMS and HRESIMS were recorded on a Bruker APEX II mass spectrometer. Column chromatography was performed on silica gel (230–400 mesh, Merck, Darmstadt, Germany). TLC was carried out on precoated Kieselgel 60 F_254_ (0.25 mm, Merck); spots were visualized by spraying with 10% H_2_SO_4_ solution, followed by heating. HPLC was performed using a system comprised of a Hitachi L-7110 pump and a Rheodyne injection port. A normal phase semi-preparative column (Supelco Ascentis^®^ Si cat#:581515-U, 250 × 21.2 mm, 5 μm) was used for HPLC.

### 3.2. Animal Material

Specimens of the octocoral *S. flexibilis* were collected by hand using scuba equipment off the coast of southern Taiwan in July, 2011, and stored in a freezer until extraction. A voucher specimen (NMMBA-TWSC-11005) was deposited in the National Museum of Marine Biology and Aquarium, Taiwan.

### 3.3. Extraction and Isolation

Sliced bodies of the soft coral *S. flexibilis* (wet weight 3.0 kg, dry weight 950 g) were extracted with ethyl acetate (EtOAc). The EtOAc layer was separated by silica gel and eluted using *n*-hexane/EtOAc in a stepwise fashion from pure *n*-hexane–100:1–pure EtOAc to yield 11 fractions, A–K. Fraction J was chromatographed on silica gel and eluted using *n*-hexane/EtOAc (stepwise, 2:1–1:1) to afford subfractions 1–10. Fractions J3, J5 and J9 were separated by normal-phase HPLC (NP-HPLC) using a mixture of *n*-hexane and acetone as the mobile phase to afford **4** (3:1, 74.3 mg), **3** (2:1, 5.6 mg) and **2** (3:1, 16.6 mg), respectively. Fraction K was further purified by NP-HPLC using a mixture of *n*-hexane and acetone as the mobile phase to afford **1** (2:1, 22.4 mg). 

Flexibilin A (**1**): yellowish oil; 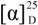 −9 (*c* 0.47, CHCl_3_); IR (neat) ν_max_ 3750–2400 (br.), 3419, 1711 cm^−1^; ^1^H (CDCl_3_, 500 MHz) and ^13^C (CDCl_3_, 125 MHz) NMR data—see Table 1; ESIMS: *m/z* 375 (M + Na)^+^; HRESIMS: *m/z* 375.2144 (calcd. for C_20_H_32_O_5_Na, 375.2147).

Flexibilin B (**2**): colorless oil; 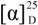 +29 (*c* 0.83, CHCl_3_); IR (neat) ν_max_ 3700–2400 (br.), 3447, 1713 cm^−1^; ^1^H (CDCl_3_, 500 MHz) and ^13^C (CDCl_3_, 125 MHz) NMR data—see Table 2; ESIMS: *m/z* 373 (M + Na)^+^; HRESIMS: *m/z* 373.1989 (calcd. for C_20_H_30_O_5_Na, 373.1991).

Flexibilin C (**3**): white powder; mp 95–97 °C; 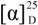 +4 (*c* 0.28, CHCl_3_); IR (neat) ν_max_ 3431, 1711 cm^−1^; ^1^H (CDCl_3_, 500 MHz) and ^13^C (CDCl_3_, 125 MHz) NMR data—see Table 3; ESIMS: *m/z* 373 (M + Na)^+^; HRESIMS: *m/z* 373.1989 (calcd. for C_20_H_30_O_5_Na, 373.1991). 

(−)-Sandensolide (**4**): white powder; mp 178–180 °C; 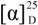 −43 (*c* 0.67, CHCl_3_) (reference [23], 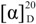 −45.0 (*c* 0.5, CHCl_3_)); IR (neat) ν_max_ 3431, 1691 cm^−1^; ^1^H NMR (CDCl_3_, 400 MHz) *δ*_H_ 6.24 (1H, s, H-17), 5.49 (1H, s, H-17′), 5.39 (1H, m, H-11), 5.36 (1H, m, H-9), 4.18 (1H, dd, *J* = 11.2, 4.4 Hz, H-13), 3.77 (1H, d, *J* = 10.4 Hz, H-5), 3.13 (1H, ddd, *J* = 13.2, 11.6, 10.4 Hz, H-10), 2.40 (1H, m, H-10′), 2.21 (1H, m, H-7), 2.13 (1H, m, H-3), 2.05 (1H, m, H-7′), 2.04 (1H, m, H-2), 2.01 (1H, m, H-1), 1.99 (1H, m, H-6), 1.80 (1H, m, H-14), 1.72 (1H, m, H-14′), 1.70 (1H, m, H-3′), 1.62 (3H, s, H_3_-18), 1.52 (3H, s, H_3_-19), 1.35 (1H, dddd, *J* = 14.0, 10.4, 3.6, 3.6 Hz, H-6′), 1.26 (3H, s, H_3_-20), 1.14 (1H, m, H-2′); ^13^C NMR (CDCl_3_, 100 MHz) *δ*_C_ 169.6 (C-16), 144.6 (C-15), 134.2 (C-8), 132.7 (C-12), 127.9 (CH-11), 124.7 (CH-9), 124.4 (CH_2_-17), 86.6 (C-4), 76.6 (CH-13), 67.4 (CH-5), 38.0 (CH_2_-14), 34.8 (CH_2_-7), 33.7 (CH-1), 32.0 (CH_2_-3), 29.2 (CH_2_-2), 26.8 (CH_2_-10), 26.6 (CH_2_-6), 22.8 (CH_3_-18), 14.9 (CH_3_-19), 9.3 (CH_3_-20); ESIMS: *m/z* 357 (M + Na)^+^; HRESIMS: *m/z* 357.2040 (calcd. for C_20_H_30_O_4_Na, 357.2042).

### 3.4. Single-Crystal X-ray Crystallography of *(−)*-Sandensolide *(**4**) [25]*

Suitable colorless prisms of **4** were obtained from a solution of ethyl acetate. Crystal data and experimental details: C_20_H_30_O_4_, *M_r_* = 334.44, crystal size 0.25 × 0.20 × 0.20 mm, crystal system monoclinic, space group *P*2_1_ (#4), with *a* = 9.0281(3) Å, *b* = 22.2200(8) Å, *c* = 9.3289(3) Å, β = 100.547(1)°, *V* = 1839.80(11) Å,^3^, *Z* = 4, *D*_calcd_ = 1.207 g/cm^3^ and λ (Cu, kα) = 1.54178 Å. Intensity data were measured on a Bruker APEX-II CCD diffractometer equipped with a micro-focus Cu radiation source and Montel mirror up to *θ*_max_ of 26° at 100 K. All 5454 reflections were collected. The structure was solved by direct methods and refined by a full-matrix least-squares procedure. The refined structural model converged to a final *R*1 = 0.0294, *wR*2 = 0.0759 for 5398 observed reflection [*I* > 2σ(*I*)] and 455 variable parameters. The absolute configuration was determined by Flack’s method, with the Flack’s parameter determined as 0.06(10) [26].

### 3.5. Generation of Superoxide Anions and Release of Elastase by Human Neutrophils

Human neutrophils were obtained by means of dextran sedimentation and Ficoll centrifugation. Measurements of superoxide anion generation and elastase release were carried out according to previously described procedures [27,28,29,30,31,32,33]. Briefly, superoxide anion production was assayed by monitoring the superoxide dismutase-inhibitable reduction of ferricytochrome *c*. Elastase release experiments were performed using MeO-Suc-Ala-Ala-Pro-Valp-nitroanilide as the elastase substrate.

## 4. Conclusions

Cembrane-type diterpenoids are the major component of the organic extract of the soft coral, *Sinularia flexibilis*, collected from the waters of Taiwan, and compounds of this type have been shown to have the potential for use in medical applications. Our studies of *S. flexibilis* have led to the isolation of three new cembranes, flexibilins A–C (**1**–**3**), along with a known metabolite, (−)-sandensolide (**4**), and flexibilin B (**2**) displayed a moderate inhibitory effect on the release of elastase by human neutrophils. In addition to marine organisms, cembrane-type diterpenoids are also found in higher plants and bryophytes [34,35,36,37,38,39,40,41,42,43]. However, the carboxylic acid moieties exhibited in cembranes **1** and **2** are rarely found [35,44]. The soft coral, *S. flexibilis*, has begun to be transplanted to culturing tanks with a flow-through seawater system located in the National Museum of Marine Biology and Aquarium, Taiwan, for exhibition and the extraction of additional natural products in order to establish a stable supply of bioactive material.
